# AUTISM SPECTRUM DISORDER: A SYSTEMATIC REVIEW ABOUT NUTRITIONAL INTERVENTIONS

**DOI:** 10.1590/1984-0462/2020/38/2018262

**Published:** 2020-03-16

**Authors:** Manuela Albernaz Monteiro, Andressa Assumpção Abreu dos Santos, Lidiane Martins Mendes Gomes, Rosane Valéria Viana Fonseca Rito

**Affiliations:** a Universidade Federal Fluminense, Niterói, RJ, Brazil.

**Keywords:** Autistic disorder, Autism, Diet therapy, Child, Adolescent, Review, Transtorno do Espectro Autista, Autismo, Dietoterapia, Criança, Adolescente, Revisão

## Abstract

**Objective:**

To identify and analyze the scientific evidence of nutritional interventions performed in children and adolescents with Autism Spectrum Disorder.

**Data sources:**

A systematic review was conducted in the MEDLINE, Cochrane Library, Embase, LILACS, Google Scholar, PubMed, PsycINFO and Periódicos CAPES databases, using a search strategy to identify studies published between January 2003 and March 2018, in Portuguese, English and Spanish. Were included studies that described nutritional interventions in children and adolescents with autism spectrum disorders and assessed autistic behavior and/or gastrointestinal symptoms. We excluded other review articles and studies that did not include a control group in the research design. The studies were reviewed for descriptive information, and the quality of evidence was assessed through the GRADE system.

**Data synthesis:**

18 studies were included in the review, being 16 randomized clinical trials, 1 case-control study and 1 open-label trial. As a result, the implementation of a gluten-free and casein-free diet was the most used intervention among the studies. Of the total, 10 studies showed a positive association of intervention with the evaluated results, while 8 did not find of a significant association.

**Conclusions:**

Although some authors report progress in the symptoms associated with autism in individuals with Autistic Spectrum Disorder undergoing nutritional interventions, there is little scientific evidence to support the use of nutritional supplements or dietary therapies in children and adolescents with autism.

## RESUMO

**Objetivo:**

Identificar e analisar as evidências científicas de intervenções nutricionais realizadas em crianças e adolescentes com Transtorno do Espectro Autista.

**Fontes de dados:**

Realizou-se uma revisão sistemática nas bases de dados MEDLINE, Cochrane Library, Embase, LILACS, Google Acadêmico, PubMed, PsycINFO e Periódicos CAPES utilizando estratégia de busca abrangente para identificar estudos publicados entre janeiro de 2003 e março de 2018, em língua portuguesa, inglesa e espanhola. Foram incluídos estudos que descreveram intervenções nutricionais em crianças e adolescentes com Transtorno do Espectro Autista e avaliaram sintomas comportamentais e/ou sintomas gastrintestinais, sendo excluídos artigos de revisão e estudos que não incluíram um grupo controle em seu delineamento. Os estudos foram examinados para obter informações descritivas, e a qualidade de evidência foi avaliada por meio do Sistema GRADE (Grading of Recommendations Assessment, Development and Evaluation).

**Síntese dos dados:**

Dezoito estudos foram incluídos na revisão (16 ensaios clínicos randomizados, um estudo de caso-controle e um ensaio clínico aberto). As intervenções e os resultados variaram, entretanto a implementação de uma dieta livre de glúten e caseína foi a intervenção mais utilizada entre os estudos. Do total, dez estudos encontraram associação positiva entre intervenção e resultados avaliados, enquanto oito não encontraram associação significativa.

**Conclusões:**

Embora alguns autores exponham progressos nos sintomas associados ao autismo em indivíduos com esse transtorno submetidos a intervenções nutricionais, há poucas evidências científicas para apoiar o uso destas em crianças e adolescentes com autismo.

## INTRODUCTION

Autism Spectrum Disorder (ASD) is a neurodevelopmental disorder that encompasses Autism, Rett Syndrome, Asperger’s Syndrome, childhood disintegrative disorder, and global developmental disorder without further specification. Currently, 1% of the world’s population is diagnosed with ASD.^1^ In the United States, the prevalence of this disorder is one in 68 eight-year-olds.^2^

The following are established as characteristics of autism: deficits in communication and social interaction; difficulty in establishing normal conversations, whether they involve verbal or nonverbal aspects and demonstrated social interest, emotion and affection; difficulty in establishing relationships, interests and activities; insistence on doing the same things; stereotyped movements; inflexible adherence to a routine (which includes, in the nutritional field, food neophobia); and hyper or hiporeaction to sensory stimuli, including food selectivity. Currently, diagnoses are made using the Diagnostic and Statistical Manual of Mental Disorders (DSM-V), updated in 2013, since there are still no specific laboratory tests that can identify the disease.^1^ ^,^ ^3^

Little is known about the etiology and pathogenesis of ASD. Evidence suggests the involvement of diverse genetic defects in conjunction with environmental and biological factors.^4^ ^,^ ^5^ In an attempt to explain the pathophysiology involved in autism and help with making diagnoses, several studies have investigated changes in physiology and different biomarkers in subjects with ASD. Through these studies, it was observed that individuals with ASD had several biological changes, such as a greater circulation of inflammatory cytokines, modifications and nonspecific intestinal inflammation, in addition to high concentrations of amino acids and peptides from food in the blood, cerebrospinal fluid and urine, leading to a theory about the connection between autism and problems in metabolizing substances from food.^3^

Abnormalities in metabolic responses are described in studies such as the opioid excess theory first proposed by Panksepp in 1979. Such anomalies are due to the high intestinal permeability that allows harmful compounds to pass through, causing intestinal inflammation, and overrunning the blood-brain barrier, leading to changes in brain metabolism. In addition, due to the characteristic selective eating behavior of individuals with autism and how they reject certain foods, their food intake may be limited, making their intake of vitamins, minerals and essential fatty acids inadequate. This requires supplementation interventions aimed not only at improving nutritional status, but also at behavioral changes generated by nutrient deficiency.^9^ ^,^ ^10^

To date, the main treatment for ASD patients is based on pharmacotherapy, but it is still a limited resource that needs further study. In addition, the number of complementary and alternative therapies for the treatment of this disorder is increasing, and nutritional interventions are very frequent. Their goal is to minimize the deleterious effects caused by the improper metabolism of food substances.^11^ ^,^ ^12^

 This review aimed to analyze scientific evidence available in the literature and related to nutritional interventions performed in children and adolescents with ASD, in order to understand and describe the characteristics of these studies. Additionally, it aimed to evaluate the results and the relevance of existing research on the topic.

## METHOD

The study was conducted through a research strategy that considered the terms that characterize the research question structured by the Population, Intervention, Comparison and Outcome (PICO) method (Table 1). The databases used were MEDLINE, the Cochrane Library, Embase and LILACS, in addition to the Google Scholar, PubMed, PsycINFO and CAPES Periodical aggregating systems. A manual search was also performed by checking the list of “Bibliographical References” of the studies included in the review. To increase search sensitivity and ensure satisfactory search retrieval, we used, in addition to controlled vocabulary (descriptors), text words, synonyms, keywords and spelling variations, which were combined using Boolean operators. The following search terms and sequences were used: “autistic disorder” OR “autism spectrum disorder” OR “Asperger syndrome” OR “autism” OR “disorder, autistic” OR “Asperger disease” OR “Asperger disorder” AND “nutrition therapy” OR “medical nutrition therapy” OR “nutritional status” OR “nutrition” OR “diet modifications” OR “diet therapies” OR “diet, gluten-free” OR “gluten-free diet, and their respective translations into Portuguese and Spanish.

**Table 1:** Acronym for the population, intervention, comparison and outcome method.

Population	Children and adolescents with ASD.
Intervention	Nutritional modifications in the diet.
Comparison	No treatment, placebo or conventional therapies such as atypical antipsychotics, serotonin reuptake inhibitors, music therapy or other behavioral treatments.
Outcome	Changes in behavioral and gastrointestinal symptoms characteristic of individuals with ASD.
Study type	Randomized controlled trials, prospective and retrospective cohort studies, case-control and nonrandomized controlled trials.

ASD: Autism Spectrum Disorder

To be included in the systematic review, studies had to meet the following criteria:

- Include at least one person aged zero to 19 years old, diagnosed with ASD, including autism, Asperger’s Syndrome or invasive developmental disorder, not otherwise specified.
- The intervention implemented had to include diet changes of the research participants.
- The dependent variable needed to be somehow associated with behavioral symptoms of ASD and/or gastrointestinal symptoms.
- Comparative studies that included a control group.
- Original research studies that provided sufficient detail about methods and results, allowing for the identification and aggregation of data and results.
- Studies published in English, Spanish and Portuguese between January 2003 and March 2018.

Studies that were excluded were:

- Ones that evaluated children and adolescents with different developmental disorders, including ASD, and other conditions, but without separate reporting of the results.
- Ones that evaluated only surrogate outcomes (eg., plasma levels of inflammatory markers, urinary peptide excretion) or specific outcomes that are unrelated to autism symptoms and gastrointestinal symptoms.
- The procedures were implemented without the supervision or direction of researchers.

The articles found were submitted to the Mendeley website, a bibliographic reference manager, and duplicates were removed. Initially, two authors evaluated the title and abstract of the articles to determine whether they met the inclusion and exclusion criteria. The studies selected from this first analysis were independently examined by two reviewers, who read the full text.

For the articles included in the review, a clinical data extraction form was used to synthesize the following information: study design, characteristics of the population studied, description of the intervention, as well as its duration, outcome measures, evaluation tools, and qualitative and quantitative data.

The quality of evidence represents the confidence in estimating the effects presented by the studies. We used the Grading of Recommendations Assessment, Development and Evaluation (GRADE) system as an assessment tool. As such, the studies were classified into four levels: high, moderate, low and very low (A, B, C and D, respectively).^13^

The study design defined the initial classification of evidence quality, whereby evidence from randomized clinical trials started with high levels of evidence, and evidence from observational studies started with low levels of evidence. From the initial classification, aspects that could reduce or increase the level of evidence were examined. The factors responsible for the reduction in the level of evidence were: methodological limitations, inconsistency, indirect evidence, inaccuracy and publication bias. And the criteria that could raise the evidence from observational studies were: large magnitude of effect, dose-response gradient, and residual confounders.^13^ ^,^ ^14^

## RESULTS

**Figure 1:**
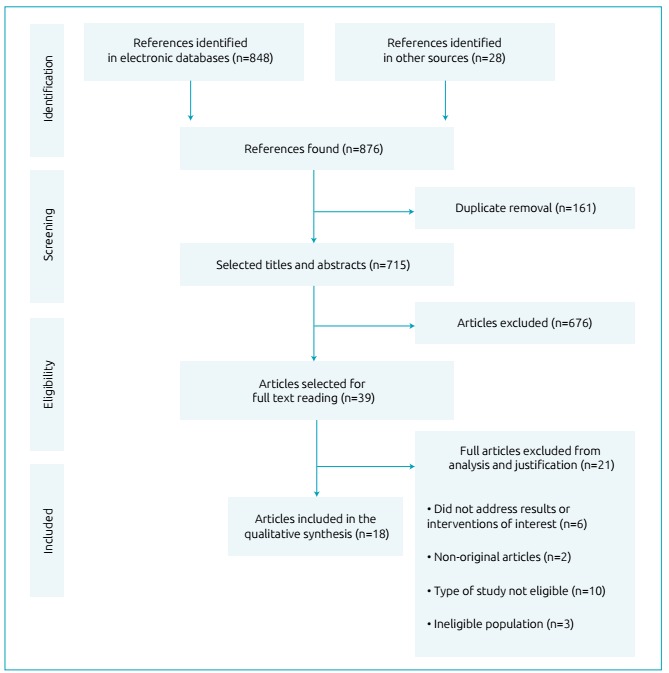
Flowchart of studies selected for review.

From the initial searches, 876 articles were selected, 848 from searches in aggregators and databases and 28 from searches from other sources. The duplicates were discarded (n = 161) and the reviewers selected articles by reading the titles and abstracts, resulting in the exclusion of 676 articles that, despite fitting the search strategy, did not address the theme studied. After the exclusion of the articles, 39 texts were read in full, and the inclusion and exclusion criteria were applied, resulting in the analysis of 18 articles for the present study (Figure 1). Of the 18 articles included, 16 were randomized controlled trials (RCT),^9^ ^,^ ^15^ ^,^ ^16^ ^,^ ^17^ ^,^ ^18^ ^,^ ^19^ ^,^ ^20^ ^,^ ^21^ ^,^ ^22^ ^,^ ^23^ ^,^ ^24^ ^,^ ^25^ ^,^ ^26^ ^,^ ^27^ ^,^ ^28^ ^,^ ^29^ and only five of them did not use the double-blind model.^19^ ^,^ ^21^ ^,^ ^24^ ^,^ ^25^ ^,^ ^29^ The total number of participants in the interventions was 639, the smallest analyzed was of 12 children^26^ and the largest was of 76.^21^ Participants’ ages ranged from two to 18 years old. Intervention time ranged from seven days to 24 months, showing a very diverse duration between interventions. Regarding the location of each study, it was observed that most were conducted in the United States (n = 9), representing 50% of the total. Four studies were conducted in Europe and the other five studies were conducted in Asia. Regarding quality, the articles were evaluated according to the GRADE System: six articles were classified in category A,^16^ ^,^ ^18^ ^,^ ^19^ ^,^ ^28^ ^,^ ^29^ ^,^ ^30^ showing low risk of bias and highly reliable evidence; ten articles were in category B, ^9^ ^,^ ^17^ ^,^ ^20^ ^,^ ^22^ ^,^ ^23^ ^,^ ^24^ ^,^ ^25^ ^,^ ^26^ ^,^ ^27^ ^,^ ^31^ with moderate risk of bias and reliable evidence; and two articles were in category C,^16^ ^,^ ^21^ indicating a high risk of bias and poor quality of evidence.

Regarding the interventions evaluated, the following were analyzed: a gluten and casein free diet; omega 3 supplements; micronutrient supplements; and alternative diets.

We found nine articles that performed nutritional interventions related to gluten and/or casein in the diet. ^20^ ^,^ ^21^ ^,^ ^23^ ^,^ ^24^ ^,^ ^25^ ^,^ ^26^ ^,^ ^27^ ^,^ ^29^ ^,^ ^31^ The Gluten Free Casein Free (GFCF) intervention was the most frequent among the studies analyzed. However, most of these studies have not shown statistical improvement regarding the clinical symptoms of autism. Some studies have shown improved communication, stereotyped movements, aggressiveness, and signs of Attention Deficit Hyperactivity Disorder, but with no statistical changes.^21^ ^,^ ^24^ ^,^ ^25^ Of these articles, one presented a different proposal, in which gluten and casein supplementation was added to the diet of children with ASD in order to evaluate maladaptive behavior. However, the results did not detect a significant change for the variable analyzed by the study.^27^ Despite having moderate evidence quality (B) and having been conducted with a restricted age group (four to seven years old), the intervention time (seven days) can be considered a bias in this sample, as pointed out by Hyman et al., in 2016, who observed improvement in symptoms after six months of this type of nutritional intervention.^23^ El-Rashidy et al. analyzed autistic behavior with the use of specific diets for six months. The patients who participated in the research were divided into three groups: the first group participated in the Atkins diet; the second, in the GFCF diet; and the third, in the normal, unrestricted diet. The first and second groups showed a significant reduction in autism scores. Scales or scores are used to assess childhood autism, with 30 being the cutoff value for ASD. A score of <30 is considered to be non-autistic; between 30 and 37 is classified as mild to moderate ASD; and between 37.5 and 60, is severe ASD. Therefore, there was a marked improvement in symptoms in the first group (Atkins diet) compared to the second group (GFCF diet).^31^ The authors pointed out that individuals under GFCF dietary intervention showed significant improvements in various aspects of development and autistic behavior, but did not specify the method used to control intake. With regard to gastrointestinal symptoms, only one study pointed to significant improvement, as the researchers themselves provided the food, in order to ensure that gluten was actually excluded from the diet.^21^ Although this study reported significant improvement in gastrointestinal symptoms, it is worth noting that the duration of this intervention was only six weeks, the age of the sample was very wide (4 to 16 years) and the quality of evidence was considered low (C).

Of the studies included in this review^16^ ^,^ ^17^ ^,^ ^18^ ^,^ ^28^ that used omega 3 supplementation as an intervention to improve the clinical condition of children and adolescents with ASD, no changes were observed in the patients. It is worth noting that the studies did not agree on the intervention time, since only one of them administered supplements for a longer period of time (six months).^28^ Another relevant aspect was the lack of consensus regarding the dosage of omega 3 supplements offered to patients. Furthermore, these studies included small samples and lacked homogeneous populations, aspects also highlighted by another review.^34^

Three articles were identified^9^ ^,^ ^22^ ^,^ ^30^that performed interventions related to micronutrient supplementation in order to improve the clinical picture of ASD. One study gave vitamin B6 and C oral supplements to the intervention group for three months. However, the authors reported general improvement in gastrointestinal symptoms in both the placebo and control groups.^9^ Another study looked at intramuscular methylcobalamin supplementation for eight weeks and found significant improvement in typical autism symptoms in the supplemented group compared with a placebo. It is worth noting that this improvement was verified in only one of the tests performed.^22^ In another survey, vitamin A oral supplements were administered for six months, with significant progress being noted in several clinical symptoms in the patients undergoing the intervention,^30^ once again reinforcing the need for a longer intervention time, one that is greater than six months.

Alternative diets were analyzed by two authors. Chan et al.^19^, in 2012, proposed a differentiated oriental diet, subjecting the intervention group to a reduction in the consumption of “hot” spices, condiments, meat and other specific foods, with significant improvement in several behavioral symptoms typical of ASD.^19^ In 2013,^15^ Al-Ayadhi et al. implemented an intervention in which cow’s milk was replaced by camel’s milk, and they reported significant improvement in communication and cognition in the two groups supplemented with camel milk, to the detriment of the cow’s milk group.^15^ It is worth noting that the different diets used foods typical of the region in question, which is a limiting factor both with regard to cost and dietary habits, when considering replicating this type of behavior in other countries, such as Brazil.

## DISCUSSION

The present review identified the most frequently implemented nutritional interventions in the treatment of children and adolescents with ASD and evaluated the quality and effectiveness of these interventions, as well as the possible limitations present in the current literature on the subject.

The use of alternative treatments for the improvement of ASD symptoms is widespread, but little evidence supports its efficacy and safety. Information from the literature in this field is very limited both in quantity and quality. Although most of the studies reported in this review found positive associations between nutritional interventions and autism symptoms, several limitations identified in the design of the research make this evidence insufficient.

A more critical analysis of each study would allow for the identification of several limitations, such as reduced sample size, the presence of heterogeneous groups, which varied in gender, age and degree of autism, as well as interventions with variable and usually short duration, not to mention the lack of pre- and post- intragroup comparisons. The use of several different methods to assess outcomes led to a lack of standardization of the studies, which also made it difficult to validate the effectiveness of the approaches.

Another issue to be addressed is the risk of confusion bias present in some articles, in which the assessments of behavioral variables and the effects of interventions were established through reports of parents, caregivers and/or teachers, which may have been distorted over time and influenced by the fact that the individuals were included in open clinical trials. Performing treatments at the same time as the clinical trial may have interfered with the results, but few studies have evaluated the efficacy/influence of these therapies. Moreover, the placebo effect may have had an impact on the results, as seen by Bent et al.^18^ In this study, the placebo group, after six weeks of study, showed improvements in hyperactivity, as assessed by the Autism behavior checklist.^18^ In 2004 Adams et al. also found progress in behavioral and gastrointestinal symptoms in the placebo group.^9^

It is worth noting that there was a lack of consensus regarding the supplement doses to be administered to the patients with ASD. Furthermore, it was not possible to establish which dosage caused symptoms to improve or at the time when the supplement should be administered in order to obtain favorable results.

These findings are in line with current reviews on the topic.^32^ In 2017, Sathe et al. systematically reviewed the effectiveness of different nutritional interventions in individuals with Autistic Spectrum Disorders and found similar results.^32^ Another systematic review investigated the effects of gluten and/or casein free diets on the treatment of autism and identified a scarcity of quality methodological evidence to support the use of this treatment in ASD.^33^

Despite the outcomes found in this study, the interventions described here are widely used in children and adolescents with ASD, by their family members - most without receiving the opinion of a clinician. Family members, caregivers and peers report visible improvement in several aspects related to the clinical and behavioral symptoms of the disorder, as well as less severe side effects compared to those triggered by drug therapy.^34^

This research did not include unpublished papers and only comparative studies containing a control group were included in the review, which may have restricted and negatively affected the number of references analyzed.

In summary, although some authors report progress in symptoms associated with autism in individuals with ASD undergoing nutritional interventions, there is insufficient scientific evidence to support its use. Therefore, studies with rigorous methodologies covering the following aspects should be developed: an intervention period of more than six months, an adequate sample size, and a well-considered set of evaluation measures and results. The aforementioned aspects will allow for a proper understanding of the consistency and precision of the impact of the intervention on these disorders, which have become an important public health issue.
